# Panobinostat synergizes with bortezomib to induce endoplasmic reticulum stress and ubiquitinated protein accumulation in renal cancer cells

**DOI:** 10.1186/1471-2490-14-71

**Published:** 2014-08-30

**Authors:** Akinori Sato, Takako Asano, Makoto Isono, Keiichi Ito, Tomohiko Asano

**Affiliations:** 1 Department of Urology, National Defense Medical College, 3-2 Namiki, Tokorozawa, Saitama 359-8513, Japan

**Keywords:** Panobinostat, Bortezomib, Endoplasmic reticulum stress, Ubiquitinated protein, Histone acetylation, Renal cancer, Combination therapy

## Abstract

**Background:**

Inducing endoplasmic reticulum (ER) stress is a novel strategy used to treat malignancies. Inhibition of histone deacetylase (HDAC) 6 by the HDAC inhibitor panobinostat hinders the refolding of unfolded proteins by increasing the acetylation of heat shock protein 90. We investigated whether combining panobinostat with the proteasome inhibitor bortezomib would kill cancer cells effectively by inhibiting the degradation of these unfolded proteins, thereby causing ubiquitinated proteins to accumulate and induce ER stress.

**Methods:**

Caki-1, ACHN, and 769-P cells were treated with panobinostat and/or bortezomib. Cell viability, clonogenicity, and induction of apoptosis were evaluated. The in vivo efficacy of the combination was evaluated using a murine subcutaneous xenograft model. The combination-induced ER stress and ubiquitinated protein accumulation were assessed.

**Results:**

The combination of panobinostat and bortezomib induced apoptosis and inhibited renal cancer growth synergistically (combination indexes <1). It also suppressed colony formation significantly (p <0.05). In a murine subcutaneous tumor model, a 10-day treatment was well tolerated and inhibited tumor growth significantly (p <0.05). Enhanced acetylation of the HDAC6 substrate alpha-tubulin was consistent with the suppression of HDAC6 activity by panobinostat, and the combination was shown to induce ER stress and ubiquitinated protein accumulation synergistically.

**Conclusions:**

Panobinostat inhibits renal cancer growth by synergizing with bortezomib to induce ER stress and ubiquitinated protein accumulation. The current study provides a basis for testing the combination in patients with advanced renal cancer.

## Background

A new therapeutic approach to advanced renal cancer is urgently needed because there is presently no curative treatment, and one innovative treatment strategy used against cancer is to induce endoplasmic reticulum (ER) stress and ubiquitinated protein accumulation [1]. Protein unfolding rates that exceed the capacity of protein chaperones cause ER stress, and chronic or unresolved ER stress can lead to apoptosis [2]. On the other hand, unfolded proteins that fail to be repaired by chaperones are then ubiquitinated and the accumulation of these ubiquitinated proteins is also cytotoxic [3].

Histone deacetylase (HDAC) 6 inhibition acetylates heat shock protein (HSP) 90 and suppresses its function as a molecular chaperon, increasing the amount of unfolded proteins in the cell [4]. Because these unfolded proteins are then ubiquitinated and degraded by the proteasome [5], HDAC6 inhibition alone is thought to cause no or only slight ER stress and ubiquitinated protein accumulation if the proteasome function is normal. We thought that combining an HDAC inhibitor with the proteasome inhibitor bortezomib would cause ER stress and ubiquitinated protein accumulation synergistically because the increased ubiquitinated proteins would not be degraded by the inhibited proteasome.

Panobinostat is a novel HDAC inhibitor that has been clinically tested not only in patients with hematological malignancies [6,7] but also patients with solid tumors, including renal cell carcinoma [8,9]. Bortezomib has been approved by the FDA and widely used for the treatment of multiple myeloma [10].

In the present study using renal cancer cells, we investigated whether the combination of panobinostat and bortezomib induces ER stress and ubiquitinated protein accumulation, and kills cancer cells effectively in vitro and in vivo.

## Methods

### Cell lines

Renal cancer cell lines (Caki-1, ACHN, and 769-P) were purchased from the American Type Culture Collection (Rockville, MD). Caki-1 cells were maintained in MEM, ACHN cells in DMEM, and 769-P cells in RPMI medium, all supplemented with 10% fetal bovine serum and 0.3% penicillin-streptomycin (Invitrogen, Carlsbad, CA).

### Reagents

Panobinostat and bortezomib were obtained from Cayman Chemical (Ann Arbor, MI) and LC Laboratories (Woburn, MA), respectively, dissolved in dimethyl sulfoxide (DMSO), and stored at -20°C until use.

### Evaluating effect of the combination of panobinostat and bortezomib on cell viability and colony formation

For cell viability assay, 5 × 10^3^ cells were plated in a 96-well culture plate one day before treatment and treated with panobinostat (25–50 nM) and/or bortezomib (5–15 nM) for 48 hours. Cell viability was evaluated by MTS assay (Promega, Madison, WI) according to the manufacturer’s protocol. For colony formation assay, 1 × 10^2^ cells were plated in 6-well plates one day before treatment and cultured for 48 hours in media containing 50 nM panobinostat and/or 10 nM bortezomib. They were then given fresh media and allowed to grow for 1–2 weeks, depending on the cell line. The number of colonies was then counted after fixing the cells with 100% methanol and staining them with Giemsa’s solution.

### Evaluating effect of the combination of panobinostat and bortezomib on induction of apoptosis

1.5 × 10^5^ cells were plated in a 6-well culture plate one day before being cultured for 48 hours in medium containing 50 nM panobinostat and/or 10 nM bortezomib. Induction of apoptosis was evaluated, using flow cytometry, by annexin-V assay and cell cycle analysis. For annexin-V assay the harvested cells were stained with annexin V according to the manufacturer’s protocol (Beckman Coulter, Marseille, France). For cell cycle analysis the harvested cells were resuspended in citrate buffer and stained with propidium iodide. They were then analyzed by flow cytometry using CellQuest Pro Software (BD Biosciences, San Jose, CA).

### Murine xenograft model

The animal protocol for this experiment has been approved by the institutional Animal Care and Use Committee of National Defense Medical College. 5-week-old male nude mice (strain BALB/c Slc-nu/nu) were purchased from CLEA (Tokyo, Japan). The animals were housed under pathogen-free conditions and had access to standard food and water ad libitum. 1 × 10^7^ Caki-1 cells were subcutaneously injected into the flank and treatments were initiated 4 days after the injection (day 1), when the tumors became palpable. The mice were divided into 4 groups of 5, the control group receiving intraperitoneal injections of DMSO and the other groups receiving either panobinostat (2 mg/kg) or bortezomib (60 μg/kg) or both. Injections were given once a day, 5 days a week, for 2 weeks. Tumor volume was estimated as one half of the product of the length and the square of the width (i.e., volume = 0.5 × length × width^2^).

### Western blotting

Cells were treated under the indicated conditions for 48 hours and whole-cell lysates were obtained using RIPA buffer. Equal amounts of protein were subjected to SDS-PAGE and transferred onto nitrocellulose membranes that were then probed with antibodies specific for glucose-regulated protein (GRP) 78, ubiquitin (Santa Cruz Biotechnology, Santa Cruz, CA), actin (Millipore, Billerica, MA), HSP70, endoplasmic reticulum resident protein (ERp) 44, endoplasmic oxidoreductin-1-like protein (Ero1-L)α, cleaved poly(ADP-ribose) polymerase (PARP) (Cell Signaling Technology, Danvers, MA), acetylated α-tubulin (Enzo Life Sciences, Farmingdale, NY), and acetylated histone (Abcam, Cambridge, UK). This probing was followed by treatment with horseradish-peroxidase-tagged secondary antibodies (Bio-Rad, Hercules, CA) and visualization by chemiluminescence (ECL, Amersham, Piscataway, NJ).

### Statistical analyses

The combination indexes were calculated using the Chou-Talalay method and CalcuSyn software (Biosoft, Cambridge, UK). The statistical significance of observed differences between samples was determined using the Mann-Whitney U test (StatView software, SAS Institute, Cary, NC). Differences were considered significant at p <0.05.

## Results

### Combination of panobinostat and bortezomib inhibited renal cancer growth synergistically

**Figure 1:**
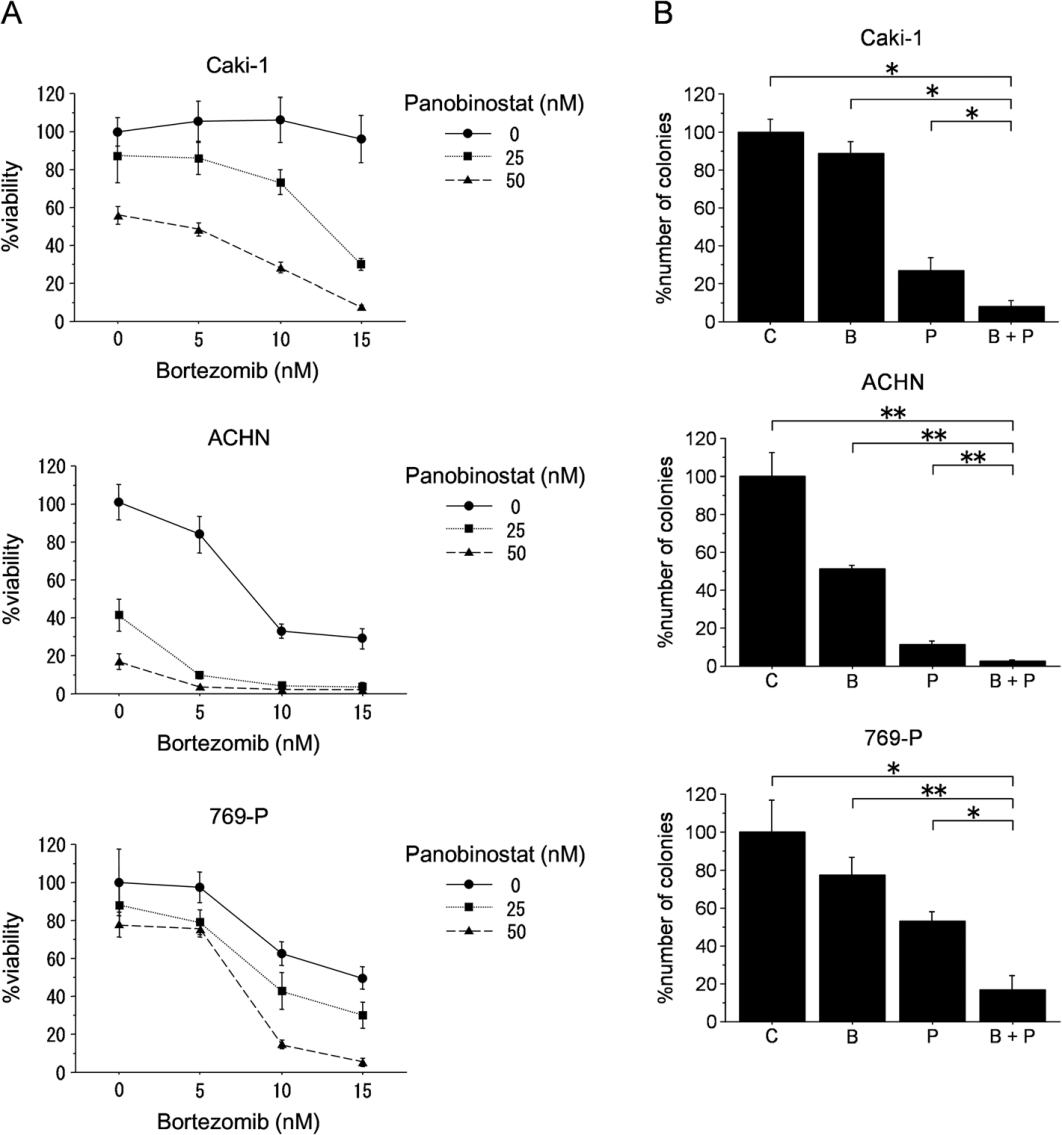
**The combination of panobinostat and bortezomib inhibited renal cancer growth effectively. A**, MTS assay results (mean ± SD, n = 6) after cells were treated for 48 hours either with bortezomib or panobinostat alone or with bortezomib and panobinostat together. **B**, Colony formation assay results (mean ± SD, n = 3) after 1–2 week incubation in control media (C) or media containing 50 nM panobinostat (P) and/or 10 nM bortezomib (B). *p = 0.0495; **p = 0.0463.

We first investigated the combined effect of panobinostat and bortezomib on renal cancer cell viability by MTS assay. Panobinostat and bortezomib each inhibited the growth of renal cancer cells in a dose-dependent fashion, and the combination did so more effectively than either did by itself (Figure 1A). Analysis using the Chou-Talalay method indicated that the effect of the combination was synergistic (combination index <1) in many of the treatment conditions (Table 1). We then investigated whether the combination affects the clonogenic survival of renal cancer cells. Colony formation assay revealed that the combination suppressed colony formation significantly and did so significantly more than did either panobinostat or bortezomib alone (Figure 1B).

**Table 1:** Combination indexes for the combination of panobinostat and bortezomib in renal cancer cells

**Panobinostat (nM)**	**Bortezomib (nM)**		
	**5**	**10**	**15**
**Caki-1**			
**25**	0.927	0.581	0.791
**50**	1.186	0.737	0.808
**ACHN**			
**25**	0.557	0.458	0.553
**50**	0.463	0.394	0.544
**769-P**			
**25**	1.074	0.803	0.946
**50**	1.41	0.512	0.519

**Figure 2:**
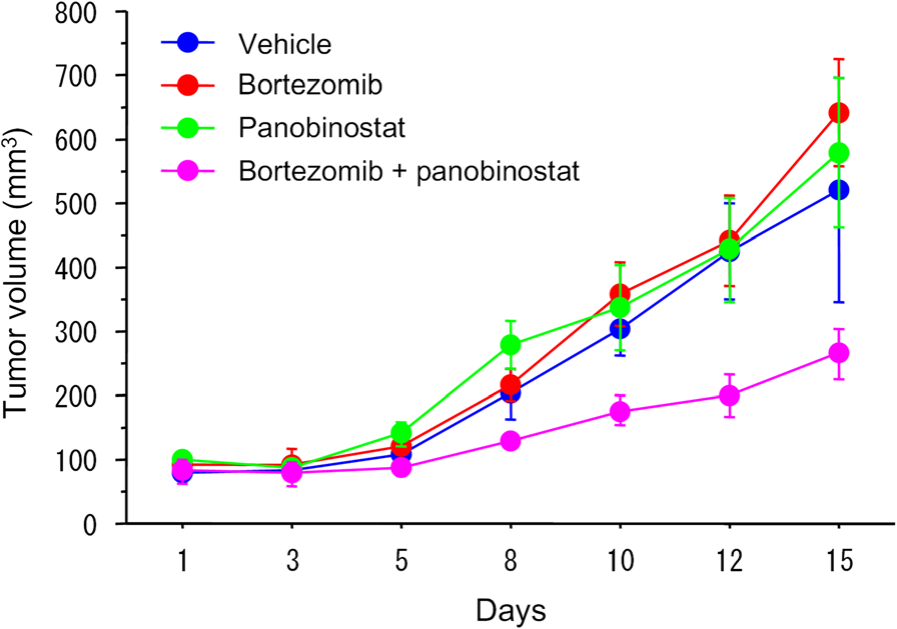
**The combination of panobinostat and bortezomib suppressed tumor growth in vivo.** A murine subcutaneous tumor model was made using Caki-1 cells, and the control group received intraperitoneal injections of DMSO, while other groups received either panobinostat (2 mg/kg) or bortezomib (60 μg/kg) or both. Injections were given once a day, 5 days a week, for 2 weeks. The 10-day treatment was well tolerated and suppressed tumor growth significantly (mean ± SE; p = 0.0283 at day 12).

We also used a subcutaneous xenograft mouse model to test the efficacy of the combination therapy in vivo. A 10-day treatment with panobinostat and bortezomib was well tolerated and suppressed tumor growth significantly (Figure 2). The p values at day 12 were 0.0283 for the control group and combination group, 0.0283 for the bortezomib group and combination group, and 0.0472 for the panobinostat group and combination group. The average tumor size at day 15 was 520 ± 175 mm^3^ (mean ± SE) in the vehicle-treated mice and was 266 ± 39 mm^3^ in the combination-treated mice. Thus the combination of panobinostat and bortezomib was shown to be effective for suppressing renal cancer growth both in vitro and in vivo.

### Combination of panobinostat and bortezomib induced apoptosis

**Figure 3:**
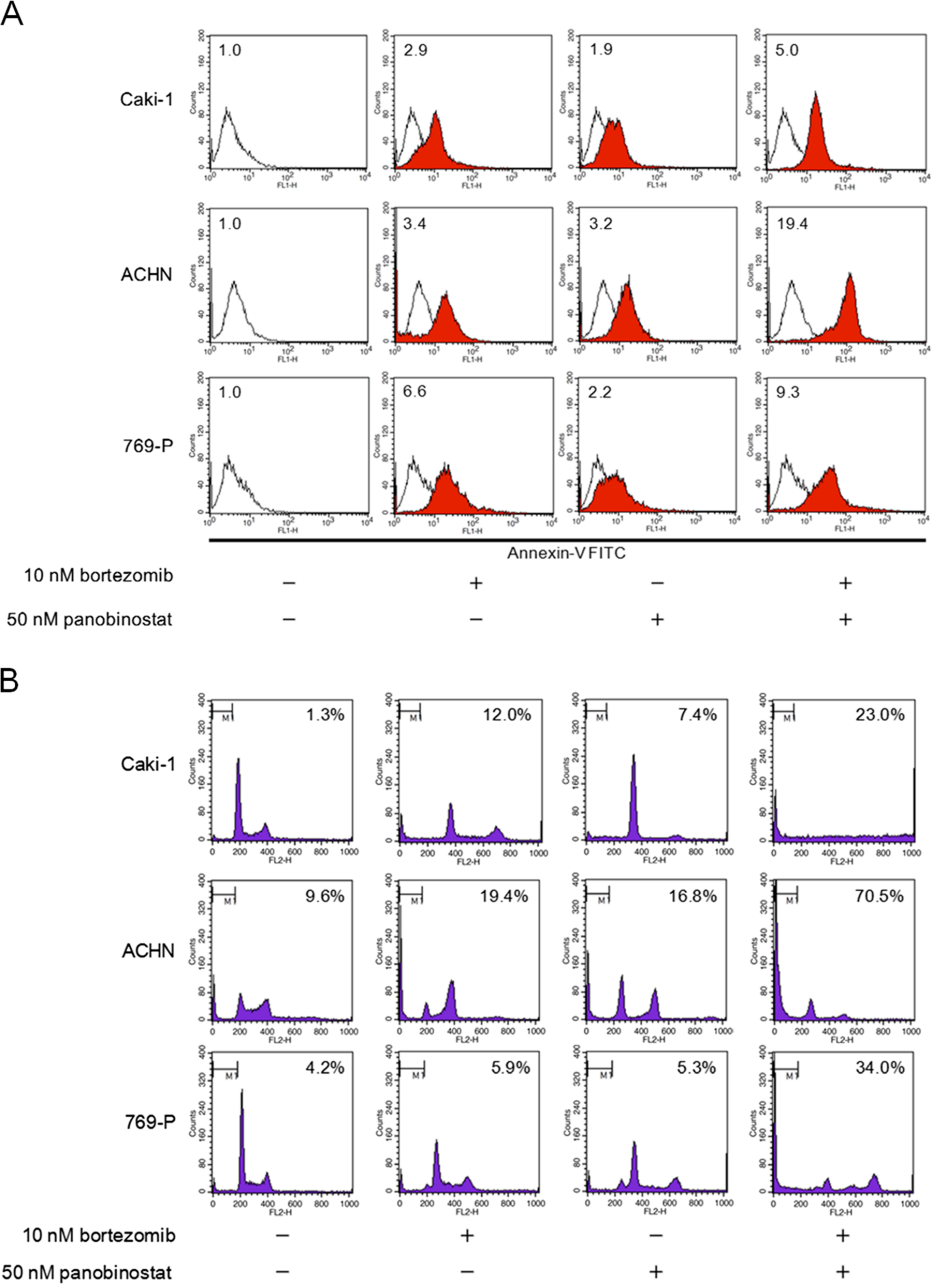
**The combination of panobinostat and bortezomib induced apoptosis in renal cancer cells.** Cells were treated for 48 hours with 50 nM panobinostat with or without 10 nM bortezomib. The combination increased the annexin-V-FITC fluorescence intensity **(A)** and increased the number of the cells in the sub-G1 fraction **(B)**. Relative annexin-V-FITC fluorescence intensity (control = 1) is shown in the insets. White, control; red, treated. The percentage of cells in the sub-G1 fraction is shown in the graph. Representative results are shown.

The combination increased the annexin-V fluorescence intensity (up to 19.4-fold compared with control vehicle) (Figure 3A) and also increased the number of the cells in the sub-G1 fraction (up to 70.5%) (Figure 3B). Thus the combination of panobinostat and bortezomib was demonstrated to induce apoptosis in renal cancer cells.

### Combination of panobinostat and bortezomib induced ER stress and ubiquitinated protein accumulation synergistically

**Figure 4:**
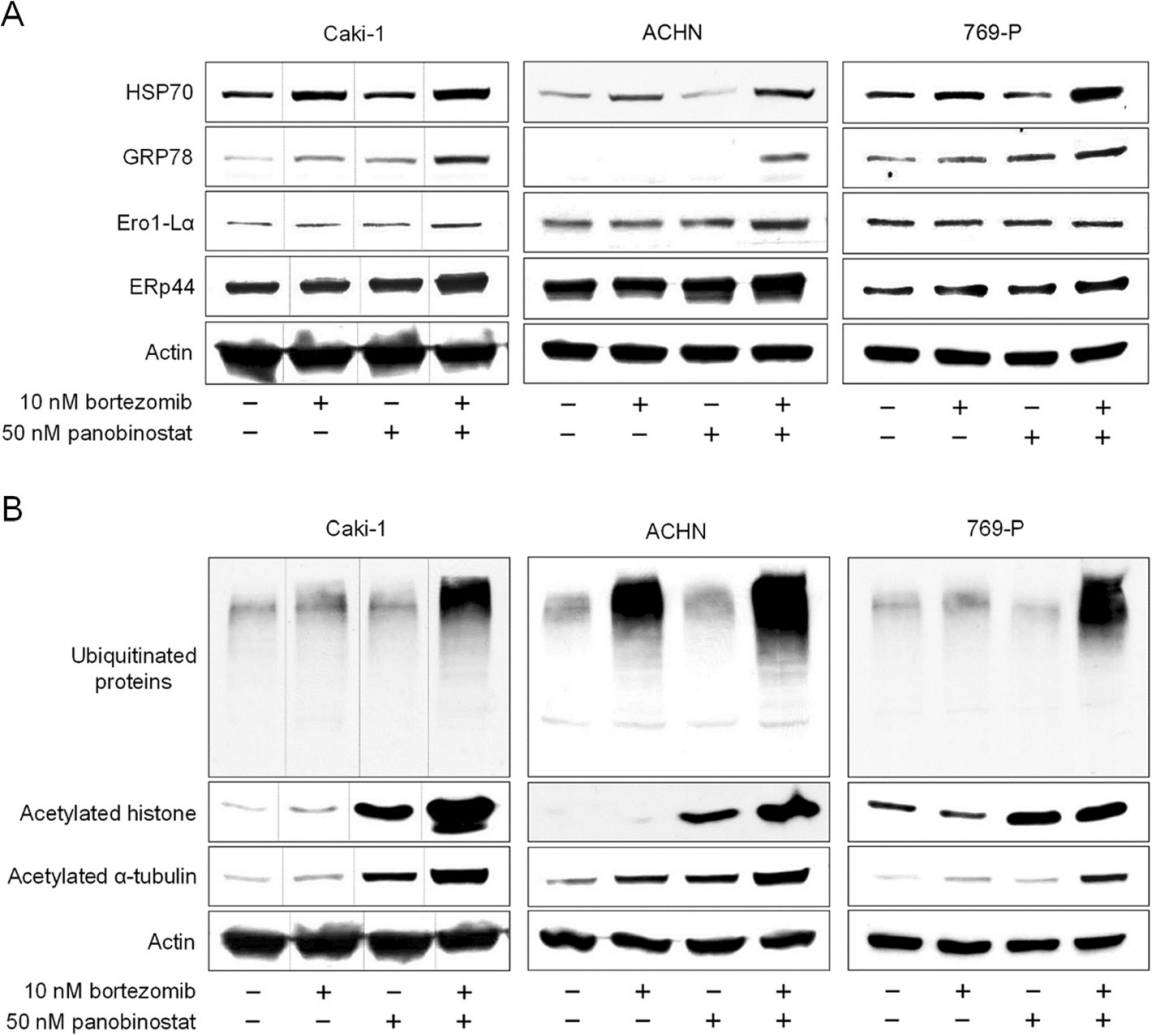
**The combination therapy induced ER stress and histone acetylation in renal cancer cells.** The 48-hour treatment with the combination of panobinostat and bortezomib induced ER stress synergistically as indicated by the increased expression of GRP78, HSP70, ERp44, and Ero1-Lα **(A)**. It also caused ubiquitinated protein accumulation in all the cell lines synergistically and enhanced histone and also α-tubulin acetylation in Caki-1 and ACHN cells. In 769-P cells, the combination enhanced the acetylation of α-tubulin but not that of histone **(B)**. The dashed lines in the Caki-1 results in parts A and B indicate that the order of the bands has been rearranged from the original gel.

The combination induced ER stress synergistically as indicated by the increased expression of ER stress markers such as GRP78, HSP70, ERp44, and (except in 769-P cells) Ero1-Lα (Figure 4A). As expected, the combination induced ubiquitinated protein accumulation synergistically (Figure 4B): in Caki-1 and 769-P cells, 10 nM bortezomib alone did not cause ubiquitinated proteins to accumulate but in combination with 50 nM panobinostat increased the accumulation of ubiquitinated proteins markedly. In ACHN cells, 10 nM bortezomib caused ubiquitinated protein accumulation and the accumulation was synergistically enhanced by 50 nM panobinostat. Acetylation of α-tubulin by panobinostat is consistent with HDAC6 inhibition because α-tubulin is one of the important substrates of HDAC6. Interestingly, the combination also enhanced the acetylation of histone and α-tubulin synergistically in Caki-1 and ACHN cells. In 769-P cells, the combination enhanced the acetylation of α-tubulin but not that of histone.

### Histone acetylation was a consequence of ubiquitinated protein accumulation

**Figure 5:**
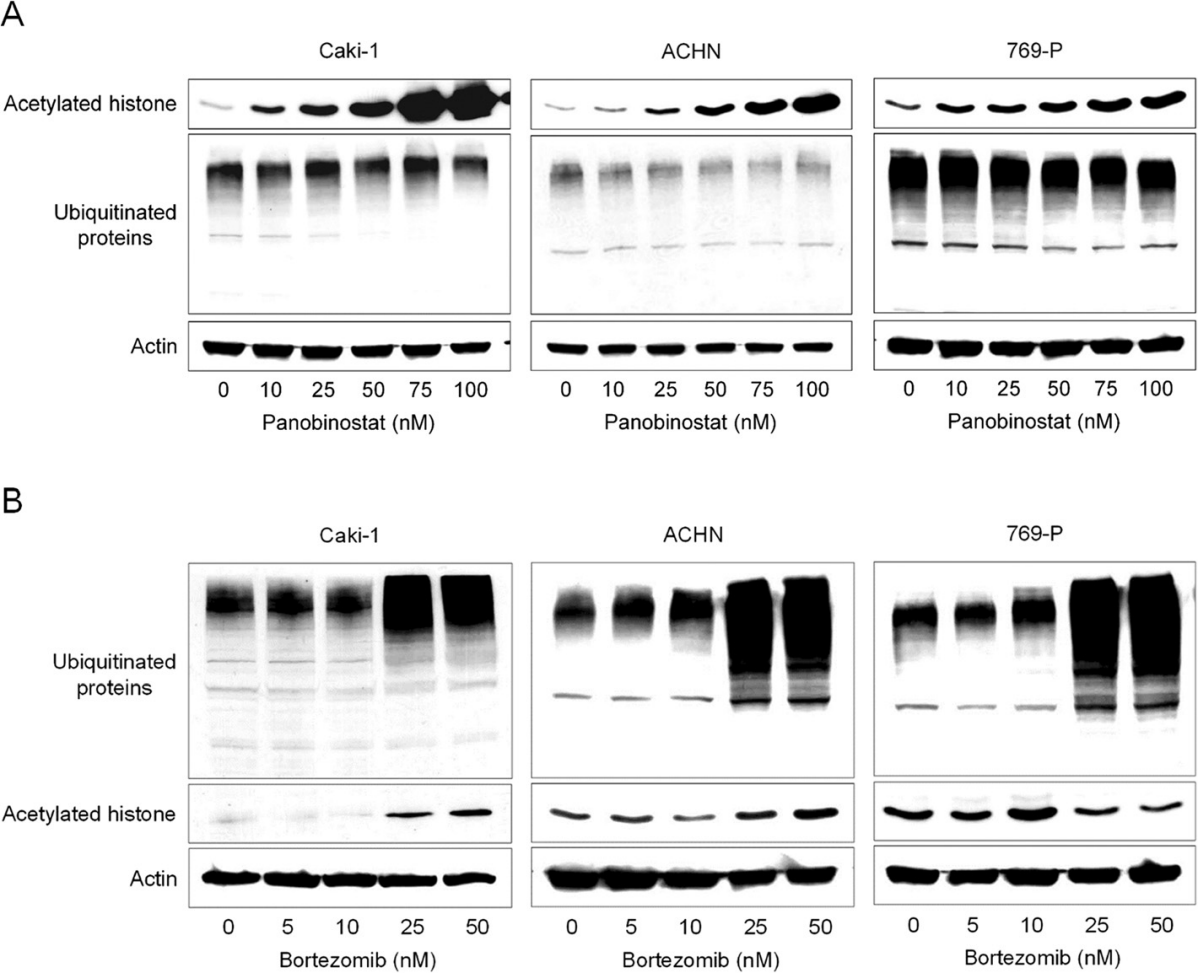
**Histone acetylation was a consequence of ubiquitinated protein accumulation. A**, 48-hour treatment with panobinostat caused dose-dependent histone acetylation in all the cell lines but did not cause ubiquitinated protein accumulation. **B**, 48-hour treatment with bortezomib, on the other hand, caused both histone acetylation and ubiquitinated protein accumulation in Caki-1 and ACHN cells and caused only ubiquitinated protein accumulation in 769-P cells.

We then investigated the relationship between histone acetylation and ubiquitinated protein accumulation. Panobinostat caused histone acetylation in a dose-dependent fashion in all the cell lines but did not induce ubiquitinated protein accumulation (Figure 5A). Bortezomib, on the other hand, caused both ubiquitinated protein accumulation and histone acetylation in a dose-dependent fashion in Caki-1 and ACHN cells but did not cause histone acetylation in 769-P cells (Figure 5B). This is in accordance with the result that the combination did not enhance histone acetylation in 769-P cells despite inducing ubiquitinated protein accumulation in them (Figure 4B). We inferred from these results that the histone acetylation the combination caused in Caki-1 and ACHN cells was a consequence of ubiquitinated protein accumulation.

## Discussion

Inducing ER stress and ubiquitinated protein accumulation is a novel approach to cancer therapy. The combination of an HDAC inhibitor and bortezomib is one of the combinations that might be expected to do it. The combination of panobinostat and bortezomib has recently been investigated mainly in hematological malignancies [11,12]. It has been reported that the combination of bortezomib and the HDAC inhibitor suberoylanilide hydroxamic acid inhibits renal cancer growth by causing accumulation of ubiquitinated proteins and histone acetylation [13], but that study did not show the relationship between ubiquitinated protein accumulation and histone acetylation. In the present study, using panobinostat, a more potent HDAC inhibitor (acting at nanomolar concentrations, whereas suberoylanilide hydroxamic acid acts at micromolar concentrations), we investigated the effect of the bortezomib-panobinostat combination on renal cancer growth as well as further mechanisms of the combination of bortezomib and an HDAC inhibitor.

Inhibition of HDAC6 acetylates HSP90, abrogating its function and increasing the amount of unfolded proteins [4]. We think that bortezomib inhibits degradation of unfolded proteins increased by panobinostat, which induces ER stress and ubiquitinated protein accumulation. Accumulation of unfolded proteins, or ER stress, activates a signaling pathway known as the unfolded protein response (UPR), which leads to increased transcription of ER folding and quality-control factors [14]. In the present study we showed the induction of ER stress by detecting the increased expression of UPR-related proteins: GRP78, HSP70, Ero1-Lα, and ERp44. GRP78 is a master regulator for ER stress because of its role as a major ER chaperone as well as its ability to control the activation of UPR signaling [15]. HSP70 is a molecular chaperone localized in the cytoplasm but associated with the regulation of the UPR by forming a stable protein complex with the cytosolic domain of inositol-requiring enzyme 1α [16]. Ero1-Lα regulates oxidative protein folding by selectively oxidizing protein disulfide isomerase [17], one of the key players in the control of disulfide bond formation [18]. ERp44 forms mixed disulfides with Ero1-Lα and may be involved in the control of oxidative protein folding [19]. The increased expression of these ER stress-related proteins thus confirmed that ER stress was induced by the combination of panobinostat and bortezomib.

Acetylation of α-tubulin, one of the important substrates of HDAC6 [20], is consistent with the inhibition of HDAC6 by panobinostat. Interestingly, panobinostat itself did not cause marked ER stress even though it inhibited HDAC6 function. This may be because the unfolded proteins increased by panobinostat can be degraded immediately by the proteasome if its function is not suppressed. This explanation is consistent with the result that panobinostat induced marked ER stress only when combined with bortezomib.

The combination induced ubiquitinated protein accumulation synergistically. This is because panobinostat increased unfolded proteins, which were then ubiquitinated, and bortezomib inhibited their degradation. The ubiquitinated protein accumulation is also in accordance with the above-discussed enhanced ER stress induced by the combination because ER stress is induced by the accumulation of unfolded proteins in the cell, and many of these unfolded proteins are ubiquitinated. Not only are ubiquitinated proteins themselves toxic to tumor cells [3], some of them may be important molecules for cancer cell survival (such as transcription factors and signal transduction molecules) that have lost their function because of unfolding and ubiquitination, presumably leading to the inhibition of multiple signal transduction pathways. Furthermore, the inhibition of NF-kB is thought to play an important role in the combination therapy with panobinostat and bortezomib because of the accumulation of undegraded IkB, a suppressor of NF-kB [21]. Jiang XJ et al. reported that the combination of panobinostat and bortezomib activated caspases and down-regulated antiapoptotic proteins such as XIAP and Bcl-2 through inhibition of the AKT and NF-kB pathways [11]. The combination is thus thought to inhibit cancer growth by diverse mechanisms other than the induction of ER stress and ubiquitinated protein accumulation.

In Caki-1 and ACHN cells the combination of panobinostat and bortezomib not only caused ubiquitinated protein accumulation but also enhanced histone acetylation. In these cell lines, panobinostat alone caused histone acetylation but not ubiquitinated protein accumulation, whereas bortezomib alone induced both ubiquitinated protein accumulation and histone acetylation. We therefore think the histone acetylation in these cell lines is a consequence of ubiquitinated protein accumulation, which is consistent with the results of a previous study using prostate cancer cells [22]. In 769-P cells, on the other hand, the combination enhanced ubiquitinated protein accumulation but not histone acetylation. This is, however, also in accordance with the result that bortezomib alone did not cause histone acetylation in 769-P cells. In Caki-1 and ACHN cells, HDAC function decreased by ubiquitination may be one explanation. In 769-P cells, bortezomib alone seems to even decrease histone acetylation. Ubiquitination may result in the HDAC activity in 769-P cells being higher than the histone acetyltransferase activity there. However, further study will be needed to clarify the exact mechanism of this decreased histone acetylation.

The combination of panobinostat and bortezomib has also been tested clinically, mainly in patients with hematological malignancies. In the most recent phase-II study enrolling 55 patients with relapsed and bortezomib-refractory myeloma [23], the patients were treated with eight 3-week cycles of 20 mg panobinostat three times a week and 1.3 mg/m^2^ bortezomib twice a week with 20 mg of dexamethasone four times a week on weeks 1 and 2. If the patients showed clinical benefit, then they were treated with 6-week cycles of panobinostat three times a week and bortezomib once a week on weeks 1, 2, and 4 with dexamethasone on the days of and after bortezomib. In that study the overall response rate was 34.5%, the clinical benefit rate was 52.7%, and grade 3 or 4 adverse events were thrombocytopenia (63.6%), fatigue (20.0%), and diarrhea (20.0%). Two limitations of our in-vivo study are that it could not provide information about whether the doses we used in mice were equivalent to those used in humans and that it lacked a proper assessment of side effects. This study is, however, the first to show the beneficial combined effect of panobinostat and bortezomib in renal cancer cells, and it provides a basis for testing the combination in clinical settings.

## Conclusions

Panobinostat inhibits renal cancer growth by synergizing with bortezomib to induce ER stress and ubiquitinated protein accumulation. Histone acetylation may be another important mechanism of action. This is the first study to demonstrate the combination’s effect on renal cancer cells both in vitro and in vivo, and it provides a basis for testing the combination in patients with advanced renal cancer.

## Competing interests

The authors declare that they have no competing interests.

## Authors’ contributions

AS contributed to design and interpretation of all experiments, drafting of the manuscript and execution of western blotting, colony formation assay and animal experiments. TA collected and assembled data and performed MTS assay, cell cycle analysis, annexin-V assay and animal experiments. MI participated in the study design, performed statistical analysis and helped to draft the manuscript. KI contributed to design and interpretation of all experiments and drafting of the manuscript. TA participated in the study design and coordination and helped to draft the manuscript. All authors read and approved the final manuscript.

## Pre-publication history

The pre-publication history for this paper can be accessed here:

http://www.biomedcentral.com/1471-2490/14/71/prepub

## References

[B1] Liu Y Ye Y Proteostasis regulation at the endoplasmic reticulum: a new perturbation site for targeted cancer therapy Cell Res 2011 21 867 883 10.1038/cr.2011.75 21537343 PMC3203708

[B2] Tabas I Ron D Integrating the mechanisms of apoptosis induced by endoplasmic reticulum stress Nat Cell Biol 2011 13 184 190 10.1038/ncb0311-184 21364565 PMC3107571

[B3] Mimnaugh EG Xu W Vos M Yuan X Isaacs JS Bisht KS Gius D Neckers L Simultaneous inhibition of hsp 90 and the proteasome promotes protein ubiquitination, causes endoplasmic reticulum-derived cytosolic vacuolization, and enhances antitumor activity Mol Cancer Ther 2004 3 551 566 15141013

[B4] Bali P Pranpat M Bradner J Balasis M Fiskus W Guo F Rocha K Kumaraswamy S Boyapalle S Atadja P Seto E Bhalla K Inhibition of histone deacetylase 6 acetylates and disrupts the chaperone function of heat shock protein 90: a novel basis for antileukemia activity of histone deacetylase inhibitors J Biol Chem 2005 280 26729 26734 10.1074/jbc.C500186200 15937340

[B5] Glickman MH Ciechanover A The ubiquitin-proteasome proteolytic pathway: destruction for the sake of construction Physiol Rev 2002 82 373 428 11917093 10.1152/physrev.00027.2001

[B6] Duvic M Dummer R Becker JC Poulalhon N Ortiz Romero P Grazia Bernengo M Lebbé C Assaf C Squier M Williams D Marshood M Tai F Prince HM Panobinostat activity in both bexarotene-exposed and -naïve patients with refractory cutaneous T-cell lymphoma: results of a phase II trial Eur J Cancer 2013 49 386 394 10.1016/j.ejca.2012.08.017 22981498

[B7] Wolf JL Siegel D Goldschmidt H Hazell K Bourquelot PM Bengoudifa BR Matous J Vij R de Magalhaes-Silverman M Abonour R Anderson KC Lonial S Phase II trial of the pan-deacetylase inhibitor panobinostat as a single agent in advanced relapsed/refractory multiple myeloma Leuk Lymphoma 2012 53 1820 1823 10.3109/10428194.2012.661175 22288662

[B8] Morita S Oizumi S Minami H Kitagawa K Komatsu Y Fujiwara Y Inada M Yuki S Kiyota N Mitsuma A Sawaki M Tanii H Kimura J Ando Y Phase I dose-escalating study of panobinostat (LBH589) administered intravenously to Japanese patients with advanced solid tumors Invest New Drugs 2012 30 1950 1957 10.1007/s10637-011-9751-0 21964801

[B9] Hainsworth JD Infante JR Spigel DR Arrowsmith ER Boccia RV Burris HA A phase II trial of panobinostat, a histone deacetylase inhibitor, in the treatment of patients with refractory metastatic renal cell carcinoma Cancer Invest 2011 29 451 455 21696296 10.3109/07357907.2011.590568

[B10] Kane RC Farrell AT Sridhara R Pazdur R United States Food and Drug Administration approval summary: bortezomib for the treatment of progressive multiple myeloma after one prior therapy Clin Cancer Res 2006 12 2955 2960 10.1158/1078-0432.CCR-06-0170 16707588

[B11] Jiang XJ Huang KK Yang M Qiao L Wang Q Ye JY Zhou HS Yi ZS Wu FQ Wang ZX Zhao QX Meng FY Synergistic effect of panobinostat and bortezomib on chemoresistant acute myelogenous leukemia cells via AKT and NF-κB pathways Cancer Lett 2012 326 135 142 10.1016/j.canlet.2012.07.030 22863538

[B12] Rao R Nalluri S Fiskus W Savoie A Buckley KM Ha K Balusu R Joshi A Coothankandaswamy V Tao J Sotomayor E Atadja P Bhalla KN Role of CAAT/enhancer binding protein homologous protein in panobinostat-mediated potentiation of bortezomib-induced lethal endoplasmic reticulum stress in mantle cell lymphoma cells Clin Cancer Res 2010 16 4742 4754 10.1158/1078-0432.CCR-10-0529 20647473 PMC2948590

[B13] Sato A Asano T Ito K Sumitomo M Asano T Suberoylanilide hydroxamic acid (SAHA) combined with bortezomib inhibits renal cancer growth by enhancing histone acetylation and protein ubiquitination synergistically BJU Int 2012 109 1258 1268 10.1111/j.1464-410X.2011.10533.x 21895936

[B14] Mori K Tripartite management of unfolded proteins in the endoplasmic reticulum Cell 2000 101 451 454 10.1016/S0092-8674(00)80855-7 10850487

[B15] Lee AS The ER chaperone and signaling regulator GRP78/BiP as a monitor of endoplasmic reticulum stress Methods 2005 35 373 381 10.1016/j.ymeth.2004.10.010 15804610

[B16] Gupta S Deepti A Deegan S Lisbona F Hetz C Samali A HSP72 protects cells from ER stress-induced apoptosis via enhancement of IRE1alpha-XBP1 signaling through a physical interaction PLoS Biol 2010 8 e1000410 10.1371/journal.pbio.1000410 20625543 PMC2897763

[B17] Mezghrani A Fassio A Benham A Simmen T Braakman I Sitia R Manipulation of oxidative protein folding and PDI redox state in mammalian cells EMBO J 2001 20 6288 6296 10.1093/emboj/20.22.6288 11707400 PMC125306

[B18] Bulleid NJ Freedman RB Defective co-translational formation of disulphide bonds in protein disulphide-isomerase-deficient microsomes Nature 1988 335 649 651 10.1038/335649a0 3173483

[B19] Anelli T Alessio M Mezghrani A Simmen T Talamo F Bachi A Sitia R ERp44, a novel endoplasmic reticulum folding assistant of the thioredoxin family EMBO J 2002 21 835 844 10.1093/emboj/21.4.835 11847130 PMC125352

[B20] Hubbert C Guardiola A Shao R Kawaguchi Y Ito A Nixon A Yoshida M Wang XF Yao TP HDAC6 is a microtubule-associated deacetylase Nature 2002 417 455 458 10.1038/417455a 12024216

[B21] Mitsiades N Mitsiades CS Poulaki V Chauhan D Fanourakis G Gu X Bailey C Joseph M Libermann TA Treon SP Munshi NC Richardson PG Hideshima T Anderson KC Molecular sequelae of proteasome inhibition in human multiple myeloma cells Proc Natl Acad Sci U S A 2002 99 14374 14379 10.1073/pnas.202445099 12391322 PMC137891

[B22] Sato A Asano T Ito K Asano T Vorinostat and bortezomib synergistically cause ubiquitinated protein accumulation in prostate cancer cells J Urol 2012 188 2410 2418 10.1016/j.juro.2012.07.108 23088964

[B23] Richardson PG Schlossman RL Alsina M Weber DM Coutre SE Gasparetto C Mukhopadhyay S Ondovik MS Khan M Paley CS Lonial S PANORAMA 2: panobinostat in combination with bortezomib and dexamethasone in patients with relapsed and bortezomib-refractory myeloma Blood 2013 122 2331 2337 10.1182/blood-2013-01-481325 23950178

